# Sex-based clinical and immunological differences across lupus erythematosus subtypes: a cross-sectional multicentre study from China

**DOI:** 10.1136/lupus-2025-001783

**Published:** 2026-02-23

**Authors:** Yu Pan, Hui Jin, Shihang Zhou, Zhang Ying, Leilei Bai, Qianjin Lu

**Affiliations:** 1 Department of Epidemiology and Biostatistics, School of Public Health, Nanjing Medical University, Nanjing, Jiangsu, China; 2 Hospital for Skin Diseases, Institute of Dermatology, Chinese Academy of Medical Sciences & Peking Union Medical College, Nanjing, Jiangsu, China; 3 Key Laboratory of Basic and Translational Research on Immune-Mediated Skin Diseases, Chinese Academy of Medical Sciences, Nanjing, Jiangsu, China; 4 Department of Dermatology and Cosmetic Medicine Center, The Second Affiliated Hospital of Chongqing Medical University, Chongqing, China; 5 Department of Infection Control and Public Health, The Second Affiliated Hospital of Jiaxing University, Jiaxing, Zhejiang, China

**Keywords:** Antibodies, Autoimmune Diseases, Autoimmunity, Lupus Erythematosus, Systemic

## Abstract

**Objective:**

To investigate sex-related differences in clinical and immunological features across lupus erythematosus (LE) subtypes.

**Methods:**

This cross-sectional analysis, based on the Lupus Erythematosus Multicenter Case–Control Study in Chinese populations (ChiCTR2100048939), included patients with SLE and major cutaneous LE (CLE) subtypes. Sex-specific comparisons were performed using R V.4.4.2.

**Results:**

In 2097 patients (1865 SLE, 1648 CLE), female predominance was observed in all subtypes, with female-to-male ratios ranging from 11.3:1 (acute CLE, ACLE) to 2.1:1 (isolated CLE, iCLE). Except for ACLE, females had earlier or similar onset than males in all other subtypes. ACLE lesions were most common in females (67%). In male patients with LE, the proportion of discoid LE (DLE) lesions was higher than female patients (31% vs 12%). Compared with males, females exhibited higher frequencies of arthritis in SLE, ACLE, DLE and chilblain LE (CHLE). In DLE, renal involvement, haematological abnormalities and serositis were more frequently observed in females. In subacute CLE (SCLE), haematological abnormalities were significantly more common in females. Additionally, non-scarring alopecia was more common in females than in males. Females had higher autoantibody positivity in iCLE and chronic CLE, with significant differences in anti-double-stranded DNA, anti-Smith, anti-U1-nuclear ribonucleoprotein and anti-ribosomal P antibodies.

**Conclusions:**

Across the subtypes, several clinical manifestations show a consistent sex distribution: ACLE lesions, arthritis, non-scarring alopecia, Raynaud’s phenomenon and autoantibodies occur more frequently in women with LE, whereas the proportions of DLE and SCLE lesions are higher in men with LE. In addition, certain features exhibit subtype-specific sex differences: among patients with SCLE, DLE and CHLE, women show a greater propensity for systemic involvement, whereas in those with SLE and ACLE, men demonstrate a higher tendency toward systemic disease.

**Trial registration number:**

ChiCTR2100048939.

**WHAT IS ALREADY KNOWN ON THIS TOPIC**

- Lupus erythematosus (LE) encompasses a broad clinical spectrum, including various subtypes of cutaneous LE (CLE), which mainly affect the mucocutaneous system, and SLE, which may involve multiple organ systems. Although numerous studies have analysed the sex-related phenotypic differences in SLE, there is a lack of research investigating such differences across the entire LE spectrum.

**WHAT THIS STUDY ADDS**

- First, based on a relatively large sample size, we systematically compared CLE and its subtypes between sexes. Second, we conducted a more detailed analysis of cutaneous and mucosal manifestations. Additionally, our study included patients with both CLE and SLE, enabling us to assess sex differences between SLE and CLE subtypes within a single population source.
- We found that across the subtypes, several clinical manifestations show a consistent sex distribution: acute CLE (ACLE) lesions, arthritis, non-scarring alopecia, Raynaud’s phenomenon and various autoantibodies are more common in women with LE, whereas the proportions of discoid LE (DLE) and subacute CLE (SCLE) lesions are higher in men with LE. In addition, certain features exhibit subtype-specific sex differences: among patients with SCLE, DLE and chilblain LE, women show a greater propensity for systemic involvement, whereas in those with SLE and ACLE, men demonstrate a higher tendency towards systemic disease.

**HOW THIS STUDY MIGHT AFFECT RESEARCH, PRACTICE OR POLICY**

- In this study, we provide a preliminary yet systematic analysis of sex-based clinical differences among CLE subtypes. These findings may promote research on lupus pathogenesis, improve diagnostic accuracy, support more precise stratified management and ultimately enhance patient outcomes.

## Introduction

 Lupus erythematosus (LE) encompasses a broad clinical spectrum, ranging from cutaneous LE (CLE), a skin-limited disease, to SLE, a multiorgan autoimmune disorder. Skin involvement is common in SLE, affecting approximately 70%–80% of patients at some point and serving as the initial manifestation in up to 25% of cases. Acute CLE (ACLE) accounts for approximately 15% of CLE cases and is closely associated with systemic disease activity, occurring in nearly 50% of patients with SLE at diagnosis. Subacute CLE (SCLE) constitutes around 8% of CLE cases and generally follows a more protracted course. Chronic CLE (CCLE) comprises more than 70% of CLE cases, with discoid LE (DLE) as the most common variant. DLE is observed in approximately 20% of patients with SLE who exhibit cutaneous manifestations. Less common CCLE forms include hypertrophic LE, tumid lupus, lupus panniculitis and chilblain LE (CHLE).^1^

LE exhibits a strong female predominance, particularly in SLE. Globally, the annual incidence of SLE is estimated at 5.14 per 100 000 persons (range: 1.4–15.13), with approximately 400 000 new cases each year. Among women, the incidence is 8.82 per 100 000 (range: 2.4–25.99), accounting for roughly 340 000 of these new cases. In contrast, the incidence among men is significantly lower at 1.53 per 100 000 (range: 0.41–4.46).^2^ In comparison, epidemiological data for CLE are relatively limited and largely derived from local or regional studies. For instance, a study from Olmsted County, Minnesota, using the Rochester Epidemiology Project reported similar overall incidence rates for CLE and SLE within the same population, although men were found to have a relatively higher incidence and prevalence of CLE compared with SLE.^3^ Similarly, a Swedish cohort study of 1088 CLE cases diagnosed between 2005 and 2007 reported an annual CLE incidence of 4.0 per 100 000, with a female-to-male ratio of 3:1.^4^ Notably, the degree of female predominance appears to be less pronounced in CLE than in SLE.

SLE and CLE are multifactorial diseases arising from complex interactions between genetic predisposition and environmental exposures.^5^ From a hormonal perspective, females exhibit increased susceptibility to SLE, partly due to elevated circulating oestrogen levels that enhance autoantibody production, whereas androgens exert immunosuppressive effects.^6^ In contrast, sex hormones appear to play a lesser role in CLE, particularly in DLE, where their influence is relatively limited.^7^ Beyond hormonal factors, X-chromosome dosage and epigenetic regulation also contribute to the sex-specific risk.^8^ Escape from X chromosome inactivation in immune cells leads to biallelic expression of key immune-related genes such as TLR7, which is implicated in lupus pathogenesis.^9^ These factors collectively contribute to the increased susceptibility and heightened immune reactivity observed in females with lupus.

Previous studies have predominantly compared sex-related clinical features in SLE, leaving CLE largely unexplored and rarely providing a systematic analysis of skin and mucosal manifestations, despite their close association with disease activity, therapeutic response and long-term prognosis.^10^ Therefore, this study aimed to analyse sex-related differences in clinical characteristics across various LE subtypes using data from 2097 patients enrolled in the Lupus Erythematosus Multicenter Case–control Study (LEMCSC) in Chinese populations. The LEMCSC dataset offers a unique opportunity to systematically investigate sex-specific characteristics across different LE subtypes within a homogeneous cohort.

## Methods

### Study design

The research was conducted as part of the LEMCSC in Chinese populations, registered under ChiCTR2100048939.^11 12^ Between December 2013 and December 2015, patients with LE were recruited from both inpatient and outpatient settings. Participants were enrolled from 29 hospitals across 15 provinces, encompassing 30 centres, including dermatology departments and, for inpatient cases, rheumatology and nephrology departments.

### Study population

To ensure comprehensive representation of all LE subtypes, patients were included if they met at least one of the following criteria: (1) individuals who satisfied either the 1997 American College of Rheumatology (ACR97) classification criteria^13^ or the 2012 Systemic Lupus International Collaborating Clinics (SLICC) criteria for SLE,^14^ with most of these participants recruited from rheumatology and nephrology departments; or (2) Individuals presenting with cutaneous manifestations characteristic of LE, who were primarily enrolled from dermatology outpatient clinics and inpatient wards. Participants were excluded if they declined to provide informed consent or if significant physical disabilities (eg, visual or auditory impairment) or severe medical conditions prevented them from completing the required assessments.

### Variables

In this study, the collected variables included demographic data, indicators of systemic involvement, cutaneous and mucosal manifestations, and laboratory parameters (autoantibody profiles). Systemic lupus involvement, including arthritis, renal impairment, haematological disorders, serositis and neurological symptoms, was determined according to the ACR97 or SLICC12 classification criteria. Skin and mucosal manifestations, covering both lupus-specific and non-specific lesions, were evaluated using the Chinese edition of the European Society of Cutaneous Lupus Erythematosus (EUSCLE) Core Set Questionnaire.^15 16^ Autoantibody analyses were performed in the clinical laboratories of the participating tertiary A-level hospitals, where standardised reagents and internationally recognised validated negative control procedures were employed.

### Data collection

Informed consent was obtained from all enrolled participants before study initiation. Data collectors, who had received both on-site and remote training provided by the Command Centre of the Second Xiangya Hospital of China, recorded study information on standardised paper forms. Each participant was examined in person by a qualified dermatologist to ensure accurate and complete documentation of skin manifestations. Clinical characteristics were collected at three stages—initial presentation, peak severity and follow-up—through direct interviews and review of medical records.

Mucocutaneous symptoms associated with LE were recorded according to a standardised guideline primarily based on the criteria of the EUSCLE Core Set Questionnaire. This manual incorporated an extensive collection of reference photographs obtained from Chinese patients with lupus. Determination of CLE subtypes was conducted under the expert guidance of Kuhn and Landmann, who were involved in the development of the EUSCLE Core Set Questionnaire.^17^ This framework aligned with the subsequent international consensus on CLE classification,^18 19^ which integrates clinical evaluation, autoantibody profiles and histopathological findings. When obvious clinical manifestations and consistent autoantibody results were present, skin biopsy was deemed unnecessary.

Among patients with CLE, 36.7% underwent skin biopsy, most of whom had negative autoantibodies or atypical clinical features. Individuals suspected of CLE but lacking pathological confirmation were excluded. To improve diagnostic consistency, a specialist consultation panel was established, enabling investigators at each site to exchange clinical photographs and seek expert advice during data collection. The panel was required to provide feedback within 24 hours to ensure accuracy of case records.

**Figure 1:**
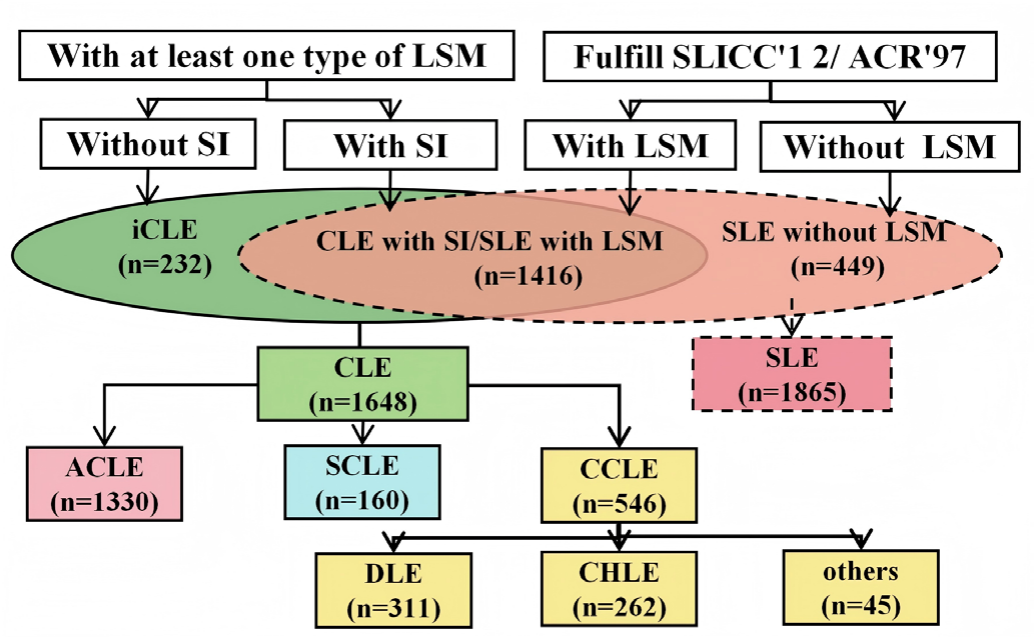
Flowchart of sample inclusion and grouping. ACLE, acute cutaneous lupus erythematosus; ACR97, 1997 American College of Rheumatology; CCLE, chronic cutaneous lupus erythematosus; CHLE, chilblain lupus erythematosus; CLE, cutaneous lupus erythematosus; DLE, discoid lupus erythematosus; iCLE, isolated cutaneous lupus erythematosus; LSM, lupus-specific cutaneous manifestations; SCLE, subacute cutaneous lupus erythematosus; SI, systemic involvement; SLICC12, 2012 Systemic Lupus International Collaborating Clinics.

All completed paper forms were returned to the central coordination unit, where they underwent quality control before being reviewed independently by two dermatologists. If the two reviewers reached agreement, the diagnosis and classification were accepted. In cases of disagreement, the records were referred to an extended panel consisting of three dermatologists and two rheumatologists, and the final diagnosis was determined once consensus was reached by at least three experts. Data entry was performed by 32 undergraduate volunteers using EpiData V.3.1, following a double-entry verification procedure to ensure reliability.^11^ Figure 1 illustrates the grouping and analysis workflow. LE profundus cases were excluded from the sex-based analyses due to the very small sample size (n=45), which we considered insufficient for meaningful comparison. CHLE cases (n=262) were retained given their relatively larger numbers, allowing for more reliable subgroup analysis.

### Statistical analysis

For a comprehensive assessment of lupus characteristics, data collected from each patient across the entire study period were used, covering the onset, peak severity and investigation stages. A positive finding at any of the three time points was regarded as positive for the entire course. Data analysis was carried out using R software V.4.4.2. Continuous variables that were not normally distributed are presented as medians with IQRs (Q1, Q3). Categorical data are shown as frequencies and percentages (%). The Kruskal-Wallis test was used to compare the distribution of non-normally distributed continuous variables. For categorical variables, the χ² test or Fisher’s exact test was used to examine association between sex and clinical manifestations. ORs were calculated to assess the strength of association between sex and clinical characteristics. Given the exploratory and descriptive nature of this study, p values <0.05 were considered suggestive evidence of sex-related differences. To account for multiple testing, a Bonferroni-adjusted significance threshold (0.05/41=0.00122) was also reported (see attachment, onlinesupplemental tables 1^8^).

### Patient and public involvement

Patients and the public were not engaged in the design, implementation, reporting or dissemination processes of this study.

## Results

### Gender differences in clinical manifestations across LE subtypes

In the Results section, we conducted a detailed analysis of the frequencies, ORs and χ² test p values of clinical manifestations between sexes across various LE subtypes (see attachment, onlinesupplemental tables 1^8^). Furthermore, we summarised the findings based on demographic characteristics, systemic involvement, cutaneous involvement and antibody profiles, with these results presented in the subsequent sections.

### Demographic characteristics of patients with different types of LE by gender

**Figure 2:**
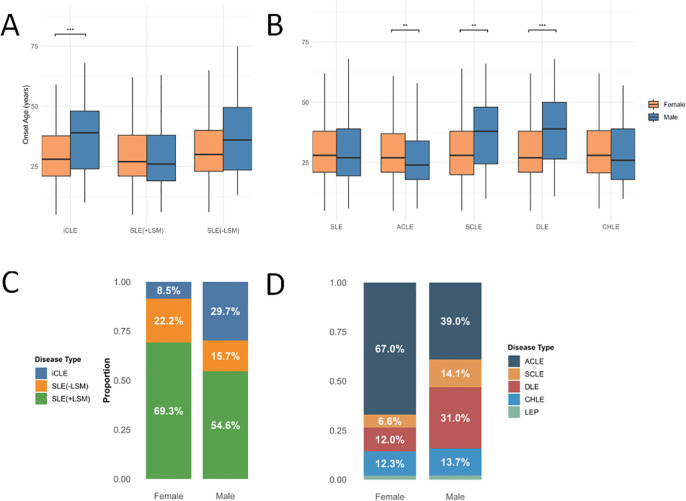
Demographic characteristics of patients with different types of LE by gender. Diseases are classified based on their spectrum, including iCLE, SLE with LSM and SLE without LSM. Traditional classification includes SLE, ACLE, SCLE, DLE and CHLE. (**A**) Comparison of the age of onset among iCLE, SLE with LSM and SLE without LSM by gender. (**B**) Comparison of the age of onset among SLE, ACLE, SCLE, DLE and CHLE by gender. (**C**) The proportion of patients with iCLE, SLE with LSM and SLE without LSM among different genders. (**D**) The proportion of patients with SLE, ACLE, SCLE, DLE and CHLE among different genders. *p<0.05, **p<0.01 and ***p<0.001. ACLE, acute cutaneous lupus erythematosus; CHLE, chilblain lupus erythematosus; DLE, discoid lupus erythematosus; iCLE, isolated cutaneous lupus erythematosus; LE, lupus erythematosus; LEP, lupus erythematosus profundus; LSM, lupus-specific cutaneous manifestations; SCLE, subacute cutaneous lupus erythematosus.

A total of 2097 patients with LE were included in this study. According to the disease spectrum classification, the patients were categorised into isolated CLE (iCLE) (n=232), SLE with lupus-specific cutaneous manifestations (SLE with LSM) (n=1416) and SLE without lupus-specific skin manifestations (SLE without LSM) (n=449). Based on the traditional classification, the patients were divided into SLE (n=1865), CLE (n=1648), ACLE (n=1330), SCLE (n=160), DLE (n=311) and CHLE (n=262). The demographic characteristics of each type of patient are detailed in table 1 and figure 2A,B.

**Table 1:** Demographic features of the population and subgroups of patients with LE

Overall patients	n	Female (n (%))	Female/male ratio	Age (median (IQR), years)	Age at onset (median (IQR), years)	Course (median (IQR), months)	Family history (n (%))
LE	2097	1848 (88.1)	7.4:1	33 (25–45)	28 (21–39)	36 (12–84)	108 (5.2)
iCLE	232	158 (68.1)	2.1:1	36 (25–47)	30 (21–43)	28 (12–69.8)	9 (3.9)
SLE	1865	1690 (90.6)	9.7:1	33 (25–45)	28 (21–39)	36 (12–84)	99 (5.3)
SLE with LSM	1416	1280 (90.4)	9.4:1	32 (24–44)	27 (21–38)	36 (12–84)	81 (5.7)
SLE without LSM	449	410 (91.3)	10.5:1	36 (26–47)	30 (23–41)	32 (8–84)	18 (4.0)
CLE	1648	1438 (87.3)	6.8:1	33 (24–44.2)	27 (21–38)	26 (12–84)	90 (5.5)
ACLE	1330	1222 (91.9)	11.3:1	32 (24–43)	27 (20–37)	36 (12–83.8)	74 (5.6)
SCLE	160	121 (75.6)	3.1:1	34 (25–47)	30 (21–40)	36 (12–86)	10 (6.3)
DLE	311	224 (72.0)	2.6:1	38 (26–47.5)	30 (22–43)	36 (14–88.5)	20 (6.4)
CHLE	262	224 (85.5)	5.9:1	33 (25–46)	28 (20–39)	52 (12–83.8)	15 (5.7)

ACLE, acute cutaneous lupus erythematosus; CHLE, chilblain lupus erythematosus; CLE, cutaneous lupus erythematosus; DLE, discoid lupus erythematosus; iCLE, isolated cutaneous lupus erythematosus; LE, lupus erythematosus; LSM, lupus-specific cutaneous manifestations; SCLE, subacute cutaneous lupus erythematosus; SLE with LSM, SLE with lupus-specific cutaneous manifestations; SLE without LSM, SLE without lupus-specific skin manifestations.

Table 1 presents the basic demographic characteristics of patients with different types of LE. In terms of disease spectrum classification, the highest female-to-male ratio was observed in SLE without LSM, with females accounting for 91.3% and a gender ratio of approximately 10.5:1. This was followed by SLE with LSM, with a sex ratio of approximately 9.4:1. The lowest ratio was found in iCLE, with females comprising 68.1% and a sex ratio of 2.1:1. According to traditional classification, the highest female-to-male ratio was observed in ACLE, with females accounting for 91.9% and a sex ratio of 11.3:1. This was followed by SLE (9.7:1), CHLE (5.9:1), SCLE (3.1:1) and DLE (2.6:1).

Among the subtypes of iCLE, ACLE, SCLE and DLE, the age of disease onset in male patients was significantly different from that of female patients (p<0.05). Specifically, male patients with iCLE, SCLE and DLE had a significantly later age of onset compared with females, while male patients with ACLE had a significantly earlier age of onset than females. No significant differences in age of onset were observed between males and females in SLE, CHLE and SLE with/without LSM (figure 2A,B).

In male patients, the proportion of iCLE, SCLE and DLE was significantly higher than that in female patients (figure 2C,D).

### Comparison of systemic involvement in patients with different types of LE by gender

**Figure 3:**
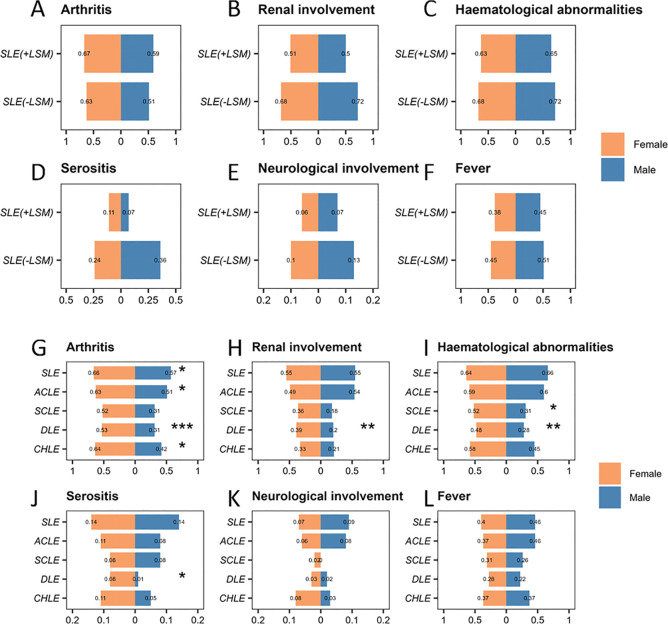
Comparison of systemic involvement in patients with different types of LE by gender. Diseases are classified based on their spectrum, including iCLE, SLE with LSM and SLE without LSM. Traditional classification includes SLE, ACLE, SCLE, DLE and CHLE. Comparison of the frequency of arthritis (**A**), renal involvement (**B**), haematological abnormalities (**C**), serositis (**D**), neurological involvement (**E**) and fever (**F**) between patients with SLE with LSM and SLE without LSM among different genders. Comparison of the frequency of arthritis (**G**), renal involvement (**H**), haematological abnormalities (**I**), serositis (**J**), neurological involvement (**K**) and fever (**L**) between patients with SLE, ACLE, SCLE, DLE and CHLE among different genders. *p<0.05, **p<0.01 and ***p<0.001. ACLE, acute cutaneous lupus erythematosus; CHLE, chilblain lupus erythematosus; DLE, discoid lupus erythematosus; iCLE, isolated cutaneous lupus erythematosus; LE, lupus erythematosus; LSM, lupus-specific cutaneous manifestations; SCLE, subacute cutaneous lupus erythematosus.

In terms of systemic involvement, including mucocutaneous involvement, arthritis, renal involvement, haematological abnormalities, serositis, neuropsychiatric involvement and fever, no statistically significant differences were observed between female and male patients across different types of LE based on disease spectrum classification (figure 3A–F).

Significant sex differences were noted in several clinical features within the traditional LE classification. In patients with SLE, arthritis was more frequent in females than in males (66.0% vs 57.1%, p=0.024). In patients with ACLE, the frequency of arthritis was significantly higher in females than in males (62.5% vs 50.9%, p=0.023). In patients with SCLE, haematological involvement was more frequent in females than in males (52.1% vs 30.8%, p=0.033). In patients with DLE, significant sex differences were observed in arthritis (52.7% vs 31.0%, p<0.001), renal involvement (38.8% vs 19.5%, p=0.002), haematological abnormalities (48.2% vs 27.6%, p=0.002) and serositis (8.0% vs 1.1%, p=0.044). In patients with CHLE, arthritis was more frequent in females than in males (63.8% vs 42.1%, p=0.018) (figure 3G–L).

Overall, female patients with LE exhibited higher frequencies of systemic involvement across various organ systems compared with male patients. Notably, females were more likely to experience arthritis. Although no statistically significant sex differences were observed, in the SLE without LSM subgroup, the proportion of renal involvement was slightly higher in males than in females, representing a numerical pattern that contrasts with the generally higher frequency of systemic manifestations in females.

### Comparison of cutaneous involvement in patients with different types of LE by gender

**Figure 4:**
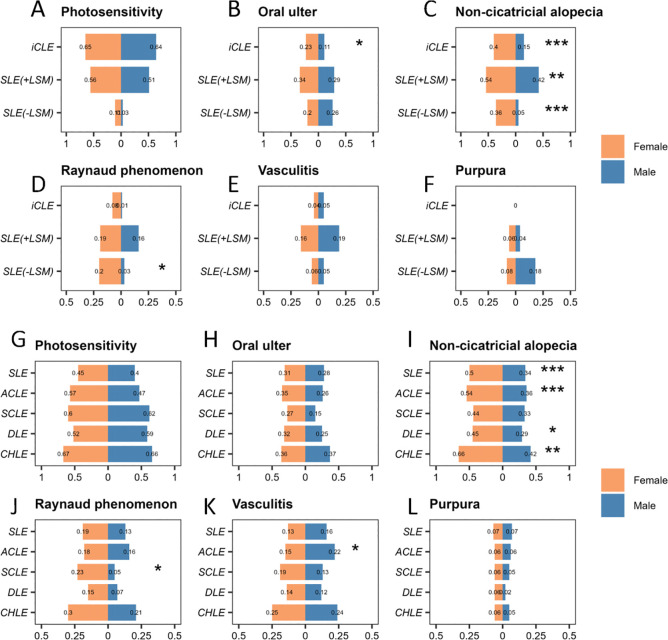
Comparison of cutaneous involvement in patients with different types of LE by gender. Diseases are classified based on their spectrum, including iCLE, SLE with LSM and SLE without LSM. Traditional classification includes SLE, ACLE, SCLE, DLE and CHLE. Comparison of the frequency of photosensitivity (**A**), oral ulcers (**B**), non-cicatricial alopecia (**C**), Raynaud phenomenon (**D**), vasculitis (**E**) and purpura (**F**) between patients with iCLE, SLE with LSM and SLE without LSM among different genders. Comparison of the frequency of photosensitivity (**G**), oral ulcers (**H**), non-cicatricial alopecia (**I**), Raynaud phenomenon (**J**), vasculitis (**K**) and purpura (**L**) between patients with SLE, ACLE, SCLE, DLE and CHLE among different genders. *p<0.05, **p<0.01 and ***p<0.001. ACLE, acute cutaneous lupus erythematosus; CHLE, chilblain lupus erythematosus; DLE, discoid lupus erythematosus; iCLE, isolated cutaneous lupus erythematosus; LE, lupus erythematosus; LSM, lupus-specific cutaneous manifestations; SCLE, subacute cutaneous lupus erythematosus.

In terms of non-specific cutaneous involvement, including photosensitivity, oral ulcers, non-scarring alopecia, Raynaud’s phenomenon, vasculitis and purpura, significant differences were observed between female and male patients in certain subtypes of LE based on disease spectrum classification. In patients with iCLE, females had a higher frequency of oral ulcers (22.8% vs 10.8%, p=0.047) and non-scarring alopecia (39.9% vs 14.9%, p<0.001) compared with males. In patients with SLE with LSM, the frequency of non-scarring alopecia was significantly higher in females than in males (54.4% vs 41.9%, p=0.007). In patients with SLE without LSM, females had a higher frequency of non-scarring alopecia (35.9% vs 5.1%, p<0.001) and Raynaud’s phenomenon (20.2% vs 2.6%, p=0.013) compared with males (figure 4A–F).

In the traditional classification of LE, significant sex differences were observed in the following non-specific cutaneous manifestations. In patients with SLE, females had a higher frequency of non-scarring alopecia compared with males (49.9% vs 33.7%, p<0.001). In patients with ACLE, females had a higher frequency of non-scarring alopecia (54.3% vs 36.1%, p<0.001) and a lower frequency of vasculitis (14.5% vs 22.2%, p=0.044) compared with males. In patients with SCLE, females had a higher frequency of Raynaud’s phenomenon compared with males (23.1% vs 5.1%, p=0.023). In patients with DLE, females had a higher frequency of non-scarring alopecia compared with males (45.1% vs 28.7%, p=0.012). In patients with CHLE, females had a higher frequency of non-scarring alopecia compared with males (66.1% vs 42.1%, p=0.008) (figure 4G–L).

### Comparison of antibody profiles in patients with different types of LE by gender

**Figure 5:**
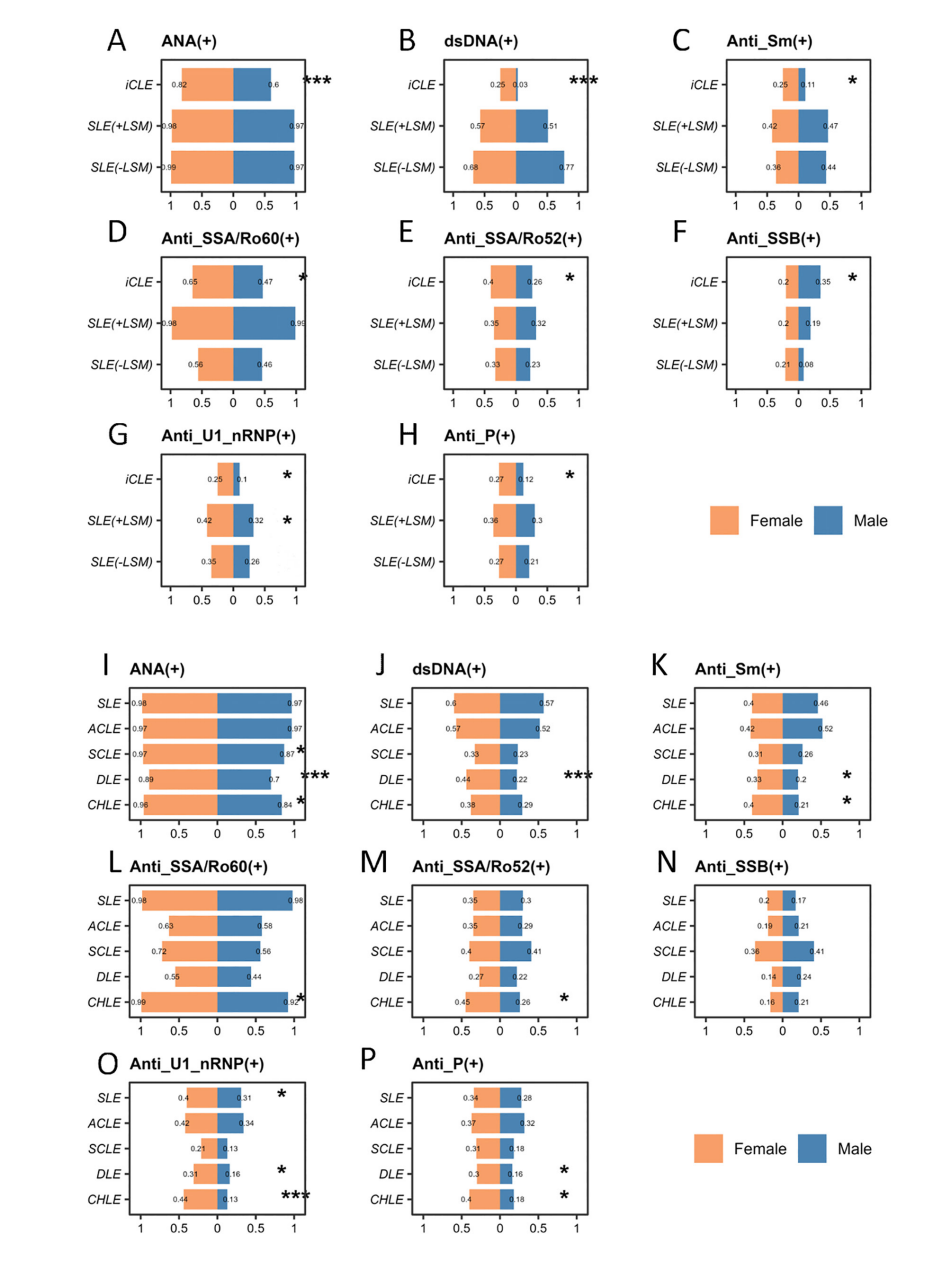
Comparison of antibody profiles in patients with different types of LE by gender. Diseases are classified based on their spectrum, including iCLE, SLE with LSM and SLE without LSM. Traditional classification includes SLE, ACLE, SCLE, DLE and CHLE. Comparison of the frequency of ANA (**A**), dsDNA (**B**), anti-Sm (**C**), anti-SSA/Ro60 (**D**), anti-SSA/Ro52 (**E**), anti-SSB (**F**), anti-U1-nRNP (**G**) and anti-P (**H**) between patients with iCLE, SLE with LSM and SLE without LSM among different genders. Comparison of the frequency of ANA (**I**), dsDNA (**J**), anti-Sm (**K**), anti-SSA/Ro60 (**L**), anti-SSA/Ro52 (**M**), anti-SSB (**N**), anti-U1-nRNP (**O**) and anti-P (**P**) between patients with SLE, ACLE, SCLE, DLE and CHLE among different genders. *p<0.05, **p<0.01 and ***p<0.001. ACLE, acute cutaneous lupus erythematosus; anti-P, antiribosomal P protein; anti-Sm, anti-Smith; anti-SSA/Ro52, anti-Sjögren’s syndrome-related antigen A/Ro52 kDa; anti-SSA/Ro60, anti-Sjögren’s syndrome-related antigen A/Ro60 kDa; anti-SSB, anti-Sjögren’s syndrome-related antigen B; anti-U1-nRNP, anti-U1 nuclear ribonucleoprotein; CHLE, chilblain lupus erythematosus; DLE, discoid lupus erythematosus; dsDNA, double-stranded DNA; iCLE, isolated cutaneous lupus erythematosus; LE, lupus erythematosus; LSM, lupus-specific cutaneous manifestations; SCLE, subacute cutaneous lupus erythematosus.

In the disease spectrum classification, female patients with iCLE exhibited significantly higher positivity rates for multiple autoantibodies compared with male patients, including: ANA (82.3% vs 59.5%, p<0.001), anti-double-stranded DNA (anti-dsDNA) antibody (24.7% vs 2.7%, p<0.001), anti-Smith (Sm) antibody (25.3% vs 10.8%, p=0.018), anti-Sjögren’s-syndrome-related antigen A/Ro60 kDa (anti-SSA/Ro60) antibody (64.6% vs 47.3%, p=0.019), anti-Sjögren’s syndrome-related antigen B (anti-SSB) antibody (19.6% vs 35.1%, p=0.017, higher in males), anti-U1 nuclear ribonucleoprotein (anti-U1-RNP) antibody (24.7% vs 9.5%, p=0.011), antiribosomal P protein (anti-P) antibody (26.6% vs 12.2%, p=0.021), anti-histone antibody (10.1% vs 0%, p=0.010) and anti-nucleosome antibody (10.8% vs 0%, p=0.008). In patients with SLE with LSM, females had a higher positivity rate for anti-U1-RNP antibody compared with males (41.7% vs 32.4%, p=0.043). In contrast, no significant differences in autoantibody positivity rates between sex were observed in patients with SLE without LSM (figure 5A–H).

In the traditional classification of LE, the following sex differences in autoantibody positivity rates were observed. In patients with SLE, females had a higher positivity rate for anti-U1-RNP antibody (40.1% vs 30.9%, p=0.021), while males had a higher positivity rate for anti-PM/Scl antibody (0.6% vs 2.3%, p=0.035). In patients with ACLE, males had higher positivity rates for anti-PM/Scl (2.8% vs 0.6%, p=0.041) and anti-Jo-1 antibodies (3.7% vs 0.7%, p=0.017). In patients with SCLE, females had a higher positivity rate for ANA (96.7% vs 87.2%, p=0.040). In patients with DLE, females had higher positivity rates for ANA (89.3% vs 70.1%, p<0.001), anti-dsDNA (43.8% vs 21.8%, p<0.001), anti-Sm (33.0% vs 19.5%, p=0.027), anti-U1-RNP (30.8% vs 16.1%, p=0.013) and anti-P antibody (29.5% vs 16.1%, p=0.023). In patients with CHLE, females had higher positivity rates for ANA (95.5% vs 84.2%, p=0.017), anti-Sm (39.7% vs 21.1%, p=0.040), anti-Sjögren’s syndrome-related antigen A/Ro52 kDa (45.1% vs 26.3%, p=0.047), anti-U1-RNP (43.8% vs 13.2%, p<0.001) and anti-P antibody (40.2% vs 18.4%, p=0.017)(figure 5I–P).

## Discussion

Interestingly, our study found that the male-to-female ratio was higher in patients with iCLE compared with those with SLE, consistent with previous epidemiological data. While SLE typically exhibits a marked female predominance with a female-to-male ratio as high as 9:1, iCLE has been reported to demonstrate a less pronounced gender disparity, with some studies observing ratios as low as 2:1 or even closer to 1:1 in population-based cohorts.^20^ This disparity may be attributable to differences in pathophysiological mechanisms. SLE is known to be strongly influenced by sex hormones, particularly oestrogens, which modulate immune responses and contribute to the heightened susceptibility in females.^21^ In contrast, cutaneous forms of lupus, particularly iCLE and DLE, are less dependent on systemic hormonal influences and more driven by localised skin immune responses, including aberrant type I interferon signalling and keratinocyte apoptosis.^5 22^ These mechanisms may reduce the sex bias observed in systemic disease. Moreover, environmental factors such as ultraviolet radiation and tobacco exposure, which have been implicated in cutaneous lupus pathogenesis, may differ in prevalence between sexes and partially account for the increased proportion of male patients in iCLE cohorts.^5 23^

In terms of systemic involvement in SLE, no significant gender differences were observed when classified by disease scope. However, traditional subtype classifications have shown that female patients exhibit significantly higher rates of manifestations such as arthritis, haematological abnormalities and serositis compared with males. This gender disparity may be closely related to the characteristics of the female immune system, which could help explain why female patients with SLE are more likely to experience multiorgan involvement. First, higher levels of oestrogen are considered a major factor contributing to the increased susceptibility of females to SLE and may also serve as one of the potential mechanisms explaining the greater propensity for multiorgan involvement in female patients with SLE compared with males.^24 25^ Additionally, genetic variations on the X chromosome are thought to be a key factor in the increased susceptibility of females to SLE, with the dual-X chromosomes in females playing a more prominent role in immune responses.^26^ In contrast, male patients with SLE without LSM and ACLE subtypes exhibit a higher proportion of renal involvement. Within the limits of our current understanding, we have not yet found other studies that report the same findings, suggesting that the absence of skin lesions may serve as a novel marker for identifying high-risk males who deserve more attention. This observation requires confirmation in larger, multicentre cohorts and the mechanisms underlying the sex difference remain to be elucidated.^27^

In terms of non-specific dermatological manifestations, our study found that female patients with SLE generally exhibit higher rates of non-scarring alopecia. Previous studies have also shown that non-scarring alopecia is more common in female patients with SLE.^28^ However, this sex difference should be interpreted with caution, as non-scarring alopecia may be under-reported in male patients due to social factors and the diagnostic challenges of distinguishing it from concomitant androgenetic alopecia. Such factors may obscure the true prevalence in males and partially contribute to the observed disparity. Additionally, the incidence of oral ulcers in female patients with iCLE, as well as the incidence of Raynaud’s phenomenon in female patients with SLE without LSM and SCLE, was significantly higher than in males. These symptoms are closely linked to immune activity and vascular reactivity, reflecting higher disease activity and greater skin and mucosal involvement in female patients.^29^ Although the occurrence of non-specific skin lesions is generally lower in male patients, the incidence of vasculitis is relatively higher, particularly in the ACLE subtype, suggesting that the vascular inflammatory mechanisms in male patients may differ from those in females, which warrants further investigation.

In terms of immunological markers, female patients exhibited significantly higher positivity rates for various autoantibodies compared with males, particularly within the iCLE subtype. Notably, however, male patients with iCLE demonstrated a statistically significant higher positivity rate for anti-SSB antibodies than females. Females consistently showed higher rates of specific antibodies such as ANA, dsDNA, anti-Sm and anti-U1-nRNP compared with males. Previous detailed comparative studies on sex-related differences in antibody profiles among LE subtypes are limited, primarily focusing on SLE. No specific studies addressing antibody profile differences by sex across CLE subtypes were found. Retrospective analyses involving 603 Brazilian patients and 107 Latin American patients with SLE revealed that males had statistically significantly higher anti-dsDNA positivity rates compared with females. Additionally, in the Brazilian cohort, male patients with SLE had higher positivity rates for anti-Sm and anti-RNP antibodies compared with females, although these differences were not statistically significant.^30 31^ Further research has used autoantibody profiles to define lupus patient subgroups, showing that patients positive for anti-dsDNA and/or anti-Sm antibodies exhibited higher frequencies of malar rash, renal and haematological involvement, and hypocomplementaemia compared with those negative for these antibodies. Similarly, anti-nRNP antibody positivity was associated with a higher prevalence of Raynaud’s phenomenon.^32^ The observed variations in antibody profiles between sexes may indicate subtype-specific immune responses, potentially contributing to the differing clinical manifestations and prognoses between males and females.

The observed gender differences suggest that personalised strategies should be considered in the diagnosis and management of LE. Female patients may require closer monitoring of immune activity-related manifestations, while male patients should be vigilant for the risk of severe organ involvement, particularly renal damage. Additionally, the gender-specific differences in antibody profiles offer potential biomarkers for accurate diagnosis and targeted therapies. Future studies should further explore the gender-related mechanisms by integrating hormonal levels, genetic backgrounds and environmental factors.

### Limitations

This study was not population-based and included a higher proportion of patients with SLE compared with those with iCLE. Nonetheless, comparing different LE subgroups provides valuable insights. The clinical manifestations and laboratory findings have been specifically evaluated in a Chinese cohort, but it remains uncertain whether these findings can be generalised to lupus patients of other ethnic backgrounds.

## Conclusions

Across the subtypes, several clinical manifestations show a consistent sex distribution: ACLE lesions, arthritis, non-scarring alopecia, Raynaud’s phenomenon and various autoantibodies occur more frequently in women with LE, whereas the proportions of DLE and SCLE lesions are higher in men with LE. In addition, certain features exhibit subtype-specific sex differences: among patients with SCLE, DLE and CHLE, women show a greater propensity for systemic involvement, whereas in those with SLE and ACLE, men demonstrate a higher tendency toward systemic disease.

## Supplementary material

10.1136/lupus-2025-001783 online supplemental table 1

10.1136/lupus-2025-001783 online supplemental table 2

10.1136/lupus-2025-001783 online supplemental table 3

10.1136/lupus-2025-001783 online supplemental table 4

10.1136/lupus-2025-001783 online supplemental table 5

10.1136/lupus-2025-001783 online supplemental table 6

10.1136/lupus-2025-001783 online supplemental table 7

10.1136/lupus-2025-001783 online supplemental table 8

10.1136/lupus-2025-001783 online supplemental table 9

## Data Availability

Data are available upon reasonable request.
